# Genome-Wide Transcription Study of *Cryptococcus neoformans* H99 Clinical Strain versus Environmental Strains

**DOI:** 10.1371/journal.pone.0137457

**Published:** 2015-09-11

**Authors:** Elaheh Movahed, Komathy Munusamy, Grace Min Yi Tan, Chung Yeng Looi, Sun Tee Tay, Won Fen Wong

**Affiliations:** 1 Department of Medical Microbiology, Faculty of Medicine, University of Malaya, Kuala Lumpur, Malaysia; 2 Department of Pharmacology, Faculty of Medicine, University of Malaya, Kuala Lumpur, Malaysia; Research Institute for Children and the Louisiana State University Health Sciences Center, UNITED STATES

## Abstract

The infection of *Cryptococcus neoformans* is acquired through the inhalation of desiccated yeast cells and basidiospores originated from the environment, particularly from bird’s droppings and decaying wood. Three environmental strains of *C*. *neoformans* originated from bird droppings (H4, S48B and S68B) and *C*. *neoformans* reference clinical strain (H99) were used for intranasal infection in C57BL/6 mice. We showed that the H99 strain demonstrated higher virulence compared to H4, S48B and S68B strains. To examine if gene expression contributed to the different degree of virulence among these strains, a genome-wide microarray study was performed to inspect the transcriptomic profiles of all four strains. Our results revealed that out of 7,419 genes (22,257 probes) examined, 65 genes were significantly up-or down-regulated in H99 versus H4, S48B and S68B strains. The up-regulated genes in H99 strain include *Hydroxymethylglutaryl-CoA synthase* (*MVA1*), *Mitochondrial matrix factor 1* (*MMF1*), *Bud-site-selection protein 8* (*BUD8*), *High affinity glucose transporter 3* (*SNF3*) and *Rho GTPase-activating protein 2* (*RGA2*). Pathway annotation using DAVID bioinformatics resource showed that metal ion binding and sugar transmembrane transporter activity pathways were highly expressed in the H99 strain. We suggest that the genes and pathways identified may possibly play crucial roles in the fungal pathogenesis.

## Introduction

Over the last decade, opportunistic fungal pathogens such as *Cryptococcus neoformans* have become increasingly important due to AIDS pandemic and the emergence of drug resistant strains [1]. *C*. *neoformans* is an encapsulated yeast-like fungus which causes life-threatening diseases of the pulmonary and central nervous system. More than 900,000 cases of cryptococcal meningitis were reported each year, resulting in approximately 600,000 deaths worldwide [2]. Cryptococcal infection is acquired by the infectious propagules [3] derived from desiccated yeast cells and basidiospores from birds excreta and decaying wood [4, 5]. Primary cryptococcal infection occurs in the lung through inhalation, causing cryptococcal pneumonia, while secondary infection due to dissemination can cause fatal meningitis/encephalitis [6].

Many studies have focused on identifying and examining the virulence factors of *C*. *neoformans* [7]. The polysaccharide capsule which increased in size during *in vivo* infection, or *in vitro* stimulation with the presence of low iron, mammalian serum, and physiological concentrations of carbon dioxide [7, 8], is a known virulence factor of *C*. *neoformans*. Additionally, growth at 37°C [9], α-mating type [10], and the production of melanin [11], phospholipase [11, 12], protein kinases [12], urease [13], cell wall integrity enzymes [14] and superoxide dismutases [15] have been documented to play a role in the invasion and survival of *C*. *neoformans in vivo* [16]. *In vivo* experiments were commonly carried out in mice model through intranasal inoculation of live yeast cells, which can result in lesions in the lung and brain tissues.

In this study, we aimed to identify novel genes which may contribute to the virulence of the *C*. *neoformans*. We first investigated the infectivity of three environmental *C*. *neoformans* isolates (H4, S48B and S68B), and the H99 reference strain, using C57BL/6 mice. Microaray analysis was then conducted to identify the differentially expressed genes among the strains, which may be responsible for cryptococcal pathogenicity.

## Materials and Methods

### 2.1. Yeasts and media

*C*. *neoformans var*. *grubii* (serotype A) H99, an isolate derived from a meningitis patient, was obtained from American Type Culture Collection (ATCC) and used as the control strain for the experiment. Three environmental strains H4, S48B and S68B were isolated from bird droppings at different locations in Klang valley, a densely populated city in the central region of Malaysia [17]. Similar to the H99 strain, all environmental strains were *C*. *neoformans* serotype A, genotype VNI with an α-mating type [18], the predominant type of *Cryptococcus* isolated worldwide [19]. All four *C*. *neoformans* strains demonstrated genetic similarity of >92.9% (Supporting Information S1 Fig).

Yeast strains were maintained at -80°C prior to the study. Cultures were streaked on the Sabouraud’s dextrose agar (SDA) and incubated at 37°C for 48 hours. To prepare cell suspension, 2 to 3 single colonies from freshly prepared plate were inoculated into Sabouraud’s dextrose broth (SDB) and incubated at 37°C for 48 hours.

### 2.2. Growth curve analysis

Three single colonies from different *C*. *neoformans* strains were inoculated separately into SDB. Cells were cultured at 37°C with agitation at 150 rpm for 108 hours. The optical density (OD) at 600 nm was measured at 6 hours intervals using a Spectronic 20 Genesys spectrophotometer (Thermo Scientific, Milford, MA). The average OD readings (mean ± SD) for each strain at different time points were calculated.

### 2.3. *In vivo* infection study

C57BL/6 mice were purchased from Jackson Laboratory (Bar Harbor, ME) and maintained in individually ventilated cages under specific pathogen free condition. Fresh culture of cryptococcal strains were washed and harvested by centrifugation at 1800 × g for 10 min. Cells were then adjusted to 10^7^ cells/ml in PBS using a heamocytometer. Mice were first anesthetized with intraperitoneal injection of a mixture ketamine (90 mg/kg) and xylazine (10 mg/kg) before inoculated with intranasal pipetting of 20 μl (2 × 10^5^ cells) yeast suspension. For fungal burden assay, mice (n = 4) were sacrificed at 20-day post-infection. Organs (lung and brain) were excised aseptically and homogenized using two glass slides in 1 ml PBS and then diluted to 10, 100 and 1000 folds. A volume of 20 μl serially diluted homogenates was plated on SDA plates and cultured at 37°C for 48 hours. Colony forming unit (CFU) per ml was determined by calculating yeast colonies on each plate. For survival study, a total of 6–9 mice were infected. All mice were examined for up to 42 days post infection. Mice were euthanized with CO_2_ inhalation to minimize suffering and distress if they exhibited severe signs including hunched posture, fur ruffling, weakness, increased respiratory rate and difficulty breathing. This study was approved by the Faculty of Medicine Ethics Committee for Animal Experimentation at the University of Malaya.

### 2.4. Antimicrobial susceptibility testing

Antimicrobial susceptibility to fluconazole (FLU) and amphotericin B (AMB) were tested using E-test strips (Liofilchem, Italy). Freshly cultured cells were adjusted to OD reading 75% transmittancy at 530 nm before spread onto SDA plate using a sterile swap. E-test strips containing AMB at concentration of 0.002 to 32 mg/l or FLU at 0.016 to 256 mg/l were then applied. The plates were incubated at 37°C for 48 hr. Minimal inhibitory concentration (MIC) values were read where the edge of the inhibition ellipse intersected with the strip.

### 2.5. Capsule induction

Yeast cells were incubated at 37°C, for 24 hours in 2 ml phosphate buffer saline (PBS) with or without 10% heat-inactivated fetal bovine serum (FBS) (Life Technologies, Rockville, MD) in six-well plates. Inactivation of sera was performed by incubation at 56°C for 30 min. To visualize the size of the capsule, a drop of India ink was added to the cell suspension on a glass slide and viewed under light microscope.

### 2.6. RNA Isolation

Yeast RNA was isolated using RNeasy mini kit (Qiagen, Valencia, CA) according to the manual instruction with minor modifications. Fresh yeast cultures at approximately 10^9^ cells were prepared and washed with cold water and harvested at 1000 ×g, 4°C for 5 minutes. The pellet was resuspended in 350 μl of lysis buffer, and distributed into two 1.5-ml microcentrifuge tubes. Then, 200 μl of 0.5 mm diameter glass beads (MoBio Laboratories, Carlsbad, CA) were added and the cells were disrupted mechanically with one cycle of agitation at 6800 rpm for 40 seconds using a homogenizer (Precellyse 24 lysis, Berlin technology, France). Samples were incubated on ice for 5 minutes and centrifuged in a microcentrifuge (Hermle, Germany) for 2 minutes at 14,000 rpm. The supernatants were transferred to a new microcentrifuge tube, mixed with 1 volume of 70% ethanol before filtering through RNeasy spin column. Column was washed and RNA was eluted using 30 μl RNase free water. The quality and quantity of the RNA were analyzed using a bioanalyzer 2100 (Agilent Technologies, Palo Alto, CA). All samples showed RNA integrity number (RIN)>7. For each sample, two biological replicates (rep1 and rep2) were prepared from two independent yeast cultures for microarray analysis.

### 2.7. Microarray

Microarray analysis was performed using customized chips (Design ID:G4102A-066930). Each microarray chip was prepared in an 8-array slide with 22,313 features per array, inclusive of the control probes. The array features were customized based on the transcript sequences available at the online BROAD Institute database (www.broadinstitute.org/annotation/genome/*Cryptococcus*_neoformans) [20]. A total of 7813 transcript sequences in fasta format were uploaded into the eArray webtool (Agilent Technologies) to design specific probes against each sequence. Three probes for each transcript were successfully designed for a total number of 7419 transcript sequences.

For microarray assay, all samples were run in duplicate and were prepared as previously described [21]. Briefly, RNA isolated from yeast cells were first labeled using a Low Input Quick Amp Labeling Kit, One-Color (Agilent Technologies). Total RNA (100 ng) was converted to double-stranded cDNA by priming with an oligo-dT primer and *in vitro* transcribed using T7 RNA polymerase to produce cyanine 3-CTP labeled cRNA. The cRNA (600 ng) was then hybridized on the slide at 10 rpm at 65°C in a hybridization oven for 17 hours. The slide was washed and scanned on a High Resolution Microarray Scanner C-model (Agilent Technologies). Raw signal data was extracted using Feature Extraction Software (V107.1.1) and data analysis was performed using a GeneSpring GX version 12.6.1 (Agilent Technologies). During data analysis, the expression filter were performed whereby probes with low raw signal intensities (<20) were excluded to eliminate noise. After filtration, data from 21,069 probes were successfully retrieved.

Hierachrical clustering was done with Euclidean distance metric and Ward’s Linkage rule clustering on both Genelist and Conditions. Predicted gene set was compared to gene ontology database by using Blast2GO software [22]. Non-matched genes were annotated using Basic Local Alignment Search Tool (BLAST) to select genes with the highest percentage of similarities. Pathway annotations of significant genes were analyzed using DAVID software [23].

### 2.8. Quantitative RT-PCR

RNA (5 μg) was reverse transcribed as previously described [24]. Quantitative real time PCR (qRT-PCR) was performed using SsoAdvanced SYBR Green Supermix (Biorad, Hercules, CA) in a Real-Time PCR 7500 (Applied Biosystems, Foster City, CA). Table 1 shows the primers used in this study. The fold change of each gene was calculated using formula (2^-ΔΔC^_T_). All samples were run in triplicates and the results were presented as mean ± SD.

**Table 1:** Primer sequences for qRT-PCR analysis. List of the forward and reverse primer sequences (5’-3’) of the up- and down-regulated genes selected for qRT-PCR analysis.

Gene Symbol	Forward primer	Reverse primer
*MMF1*	CCCTACCCAACAGTCCTCAA	CCAGCCGATTTGGAGTGTAT
*MPH2/3*	CAACAGGGTAAGTGCGGAAT	GCAGAGGACTCCAAGTCCAG
*MVA1*	TGTATGGTGAGCATGGCTGT	TGAGGAGTAGCCGAGCAAAT
*PUT3*	CGCGAGTGTTTCGCATACTA	GCGCGAGTTGATACACTTCA

### 2.9. Statistical analysis

Microarray data was analyzed using one-way analysis of variance (ANOVA) followed by Bonferroni’s post hoc test. Unpaired student’s *t*-test was used for comparison between two groups of data. Data were considered statistical significance if P < 0.05.

## Results

### 3.1. Environmental strains of *C*. *neoformans* are less virulent compared to H99

**Fig 1:**
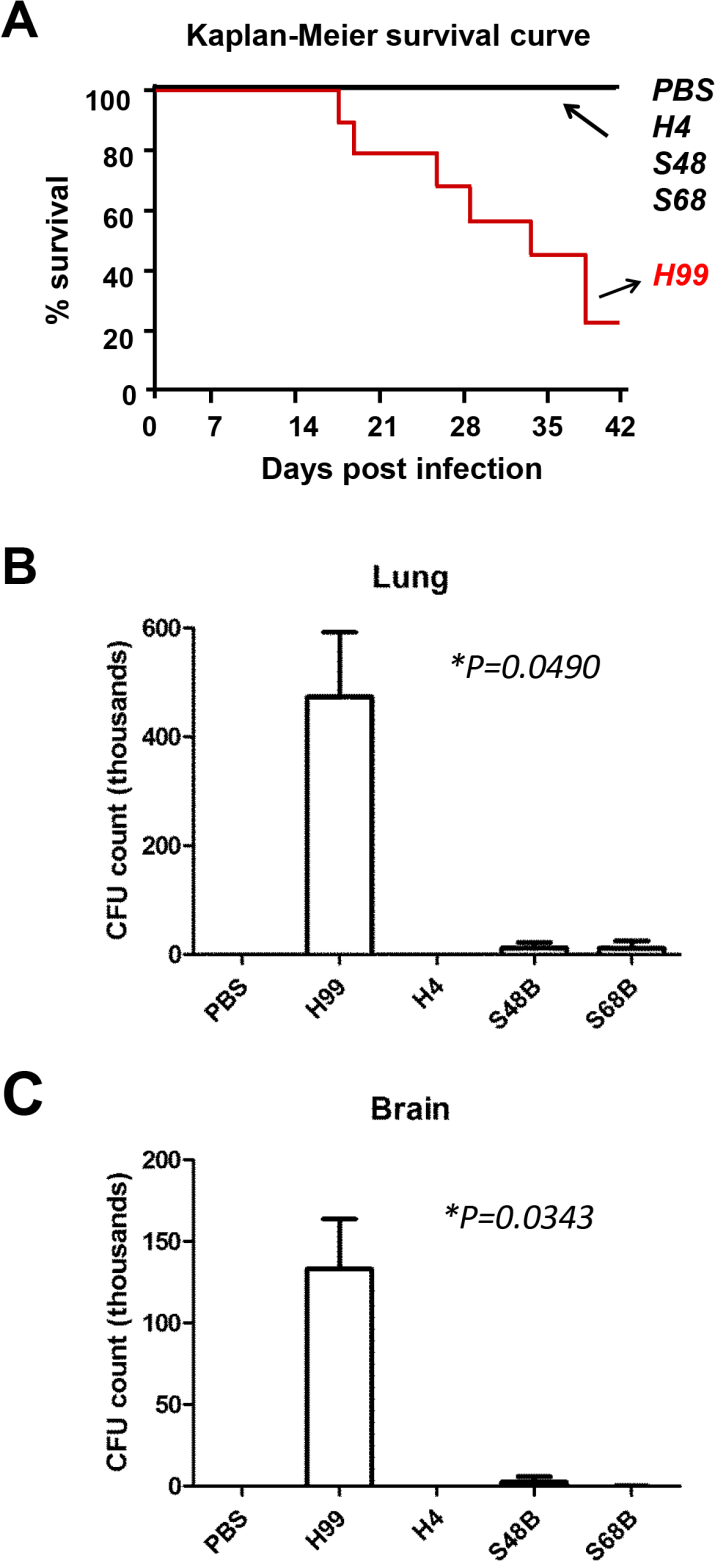
*In vivo* virulence study among different *C*. *neoformans* strains. (A) Kaplan-Meier survival curve. C57BL/6 mice at age 8–12 weeks old were administrated intranasally with 2 × 10^5^ yeast cell suspension. Mice were infected with *C*. *neoformans* clinical strain H99 or environmental strains H4, S48B and S68B, and observed for a period of 42 days. (n = 6–9). (B and C) CFU assay. Lung (B) or brain (C) homogenates from *C*. *neoformans*-infected mice (n = 4) were serially diluted and plated onto agar plates in duplicates. Numbers of colonies (CFU/ml) were counted after 48 hours. CFU formations on the plates were measured and shown as mean ± SD. Group statistical significance measured was by two-way ANOVA analysis (*P<0.05).

To compare the degree of virulence between *C*. *neoformans* H99 strain and three locally isolated environmental strains, we administrated wildtype C57BL/6 mice intranasally with yeast cells, as the respiratory tract is the portal of entry for *C*. *neoformans*. A total number of 2 × 10^5^ yeasts cells were inoculated because previous report showed that cryotococcal dissemination to the brain occurred in 100% of animals when infected at approximately 10^4^ or 10^5^ organisms, but not at lower numbers of organisms [25]. Mice infected with *C*. *neoformans* H99 strain were found dead starting from day 18, while the remaining of the mice infected with environmental strains survived throughout the end of observation period of 42 days (Fig 1A). This was in consistent with a previous study suggesting that most of the environmental isolates of *C*. *neoformans* serotype A were non-lethal to the mice [26].

At 20-day post-infection, the lung (primary infection site) and brain (secondary infection site) of the infected mice were excised, homogenized and plated for CFU counts. The fungal load in both lung and brain tissues was higher for mice infected with the H99 strain, comparing to those infected with the environmental strains (Fig 1B and 1C). The lung homogenates from the *C*. *neoformans* H99-infected mice showed an average of 4.73±1.19 × 10^5^ CFU/ml (*P* = 0.049), approximately forty folds higher than those obtained from S48B and S68B-infected mice (0.11±0.09 × 10^5^ and 0.11±0.13 × 10^5^ CFU/ml, respectively) (Fig 1B). Consistently, the brain homogenates from the *C*. *neoformans* H99-infected mice demonstrated higher CFU counts (1.33±0.31 × 10^5^ CFU/ml, *P* = 0.034) compared to those obtained from S48B and S68B-infected mice (0.02±0.03 × 10^5^ CFU and 0.01±0.006 × 10^5^ CFU/ml, respectively) (Fig 1C). No growth was obtained from the homogenates of organs excised from those mice infected with H4 environmental strain, suggesting that H4 strain was probably a non-virulent strain. Additionally, inflammation and edema were only observed from the lung tissues of mice infected with H99 strain. No specific sign of illness or pathological abnormality was observed for mice infected with the environmental strains. Hence, based on the findings obtained from the mice virulence study, we proposed that the degree of virulence for the four strains tested were H99>S48B/S68B>H4.

### 3.2. Pathogenicity of *C*. *neoformans* H99 strain was not due to extensive cell growth

**Fig 2:**
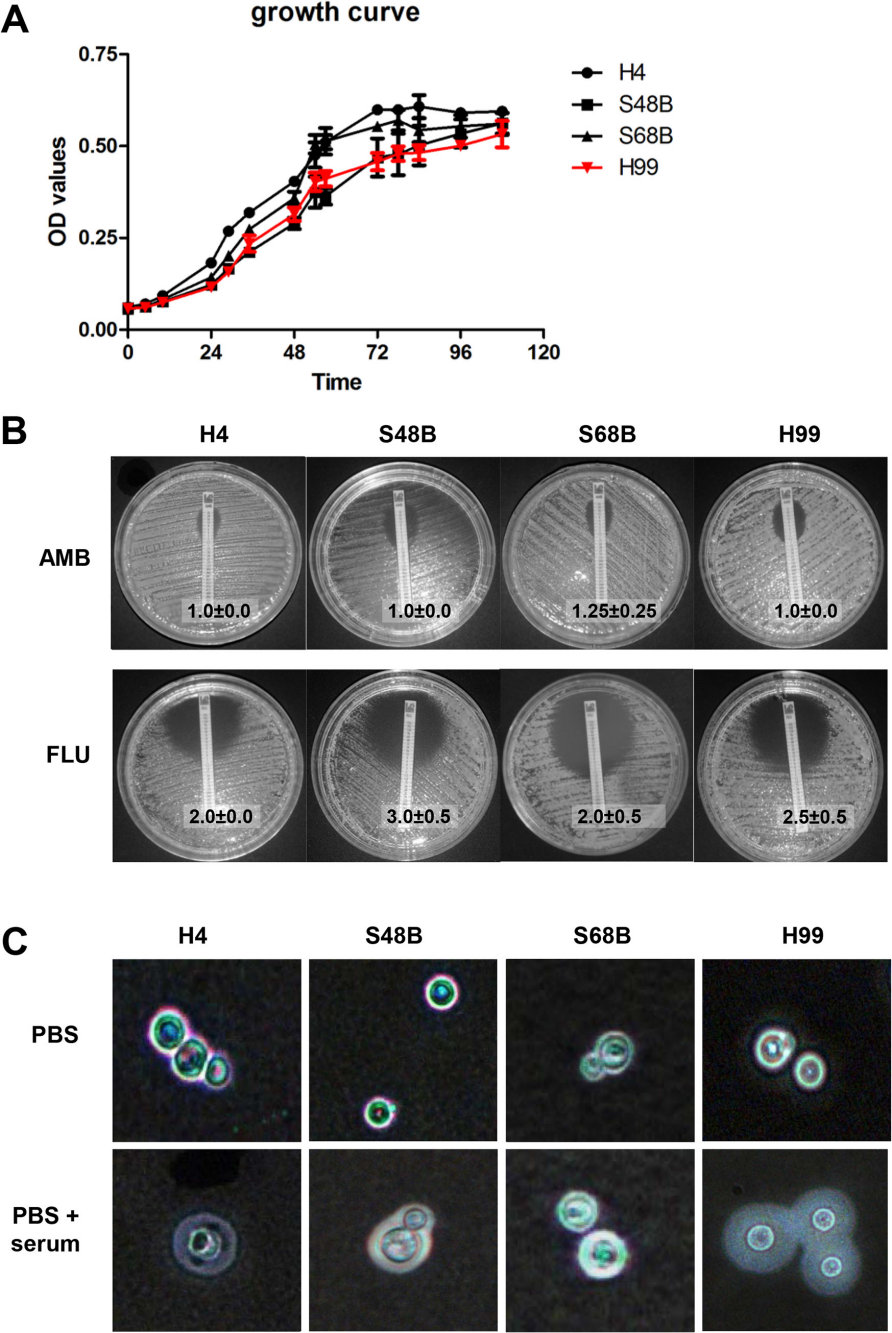
Different traits of *C*. *neoformans* strains. (A) Growth curve. *C*. *neoformans* strains (H99, H4, S48B and S68B) cells were cultured in triplicates at 37°C with gentle shaking. OD reading for each sample was measured throughout a time course of 108 hours. (B) E-test. Fungal strains were inoculated evenly onto SDA plate. An AMB or FLU-containing MIC strip was then placed on each plate and incubated at 37°C for 48 hours. Eclipse sizes which intersected with the MIC strips were recorded. Shown were mean±SD from 2 duplicate plates. Data were representative of two independent experiments. (C) Capsule formation. Fungal cells were incubated in PBS with or without the presence of 10% serum, and incubated for 24 hours at 37°C. Cells were then applied on glass slide for India ink staining.

An experiment was performed to assess the growth rate of each cryptococcal strain as the virulence of an organism may be affected by its growth rate. The real-time cell growths of the H99 compared to H4, S68B and S48 strains were determined (Fig 2A). No significant changes were observed in the growth rates of the cryptococcal strains throughout the observation period of 108 hours. However, the least virulent H4 strain demonstrated a relatively higher growth rate compared to the rest of the strains tested, followed by S48B. Meanwhile, S68B and H99 strains showed almost identical growth curve throughout the observation period. The result suggests that there is no direct link between *in vitro* growth activity in growth media and the *in vivo* pathogenicity results performed in C57BL/6 mice.

In addition to growth curve, we examined other traits of the cryptococcal strains i.e. anti-fungal resistance and capsule formation. For comparison of anti-fungal resistance among strains, an E-test was performed (Fig 2B). We observed that all 4 strains showed comparative MIC values, at 1.0 to 1.25 for AMB, and 2.0 to 3.0 for FLU, indicating strong susceptibility and no significant difference of anti-fungal resistance among H99 and environmental strains.

Capsule size is known to increase during *in vivo* infection and is associated with virulence. To examine capsule formation ability among all strains, we incubated cells in the presence of FBS at 37°C (Fig 2C). Interestingly, we observed all environmental strains were able to form capsule. Among all strains, H99 showed most prominently enlarged capsule size which may confer to strong virulence in animal study.

### 3.3. Differential expression pattern of H99 strain versus the environmental strains

**Fig 3:**
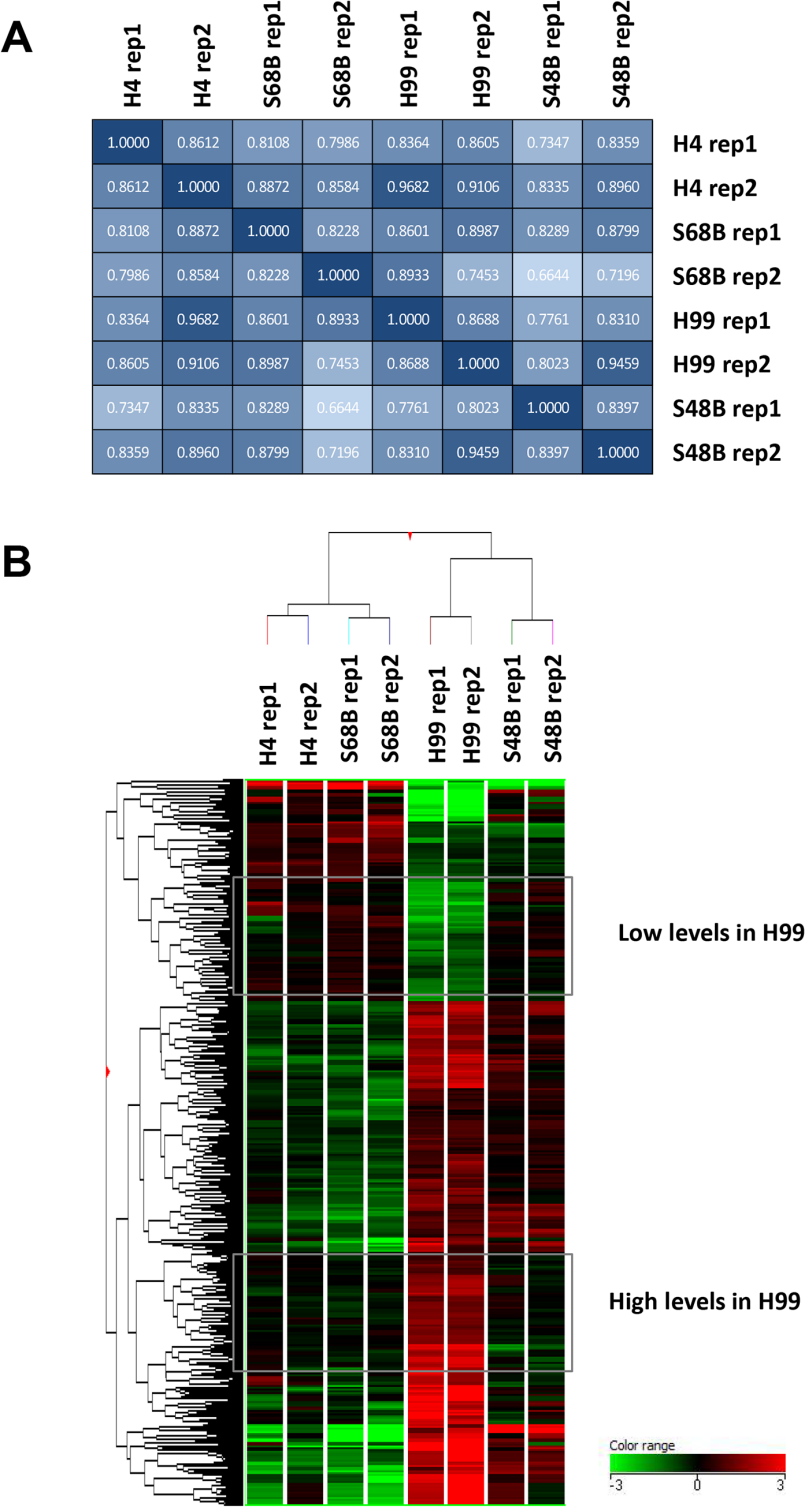
Microarray analysis. (A) Pairwise correlation matrix. Four *C*. *neoformans* strains (H99, H4, S48B and S68B) were compared. Two independent samples were prepared for each fungal strain. Rep1–2: replicates of samples. Numbers in the box represents correlation coefficient values among groups. Dark box: high correlation; light box: low correlation. (B) Gene expression heat map. Dendrogram represents the colour-coded expression levels of the significantly regulated genes. The groups of genes which showed differential expressions in H99 versus other strains were as indicated. Low levels in H99 category indicates the genes with low expression levels in H99 (green) but high expression in the environmental strains (red). In contrast, high levels in H99 category indicates the genes with high expression levels in H99 (red) but show low expression in the environmental strains (green). Hierachrical cluster was performed with Euclidean distance metric and Ward’s Linkage rule clustering. Colour range represents log_10_ (FC) of the microarray intensities.

To examine total gene expression in H99 and environmental *C*. *neoformans* strains, we isolated RNA from the fungal cells cultured at 37°C, 150 rpm (mimics the condition during *in vivo* infection) and processed for microarray analysis. Because the *C*. *neoformans* microarray chip was not commercially available, microarray slide on an 8-array chip was costumed-made. The total numbers of features analyzed on each array were 22,257 probes (7,419 genes) in triplicates. Pairwise correlation coefficient matrixes among all samples tested were as shown in Fig 3A. Duplicates of each strain (rep1 and rep2) showed correlation values which ranged from 0.8 to 1.0. The correlation among different strains were at an average of 0.8584 (0.6644 to 0.9682), suggesting that all strains shared a relatively close expression patterns. To identify differentially expressed genes, fold change (FC) of each probe was calculated by normalizing the expression level in H99 strain to the expression in environmental strains. After microarray result was filtered with FC<-2 or FC>2 (P<0.05 by Post-Hoc testing), a total of 871 probes (435 genes) were found to be significantly up- or down-regulated. Note that the number of probes was not exactly three times of the number of genes because some probes were excluded during stringent filtration process. The heatmap generated from these 871 significant probes revealed a distinct expression pattern of the H99 strain in comparison to other environmental strains (Fig 3B). Hierachrical clustering based on Euclidean distance metric and Ward’s Linkage showed closer distance of H4 to S68B, and H99 to S48B strains, consistent with molecular typing of the strains into Gr1 and Gr2 subtypes, respectively [18].

**Fig 4:**
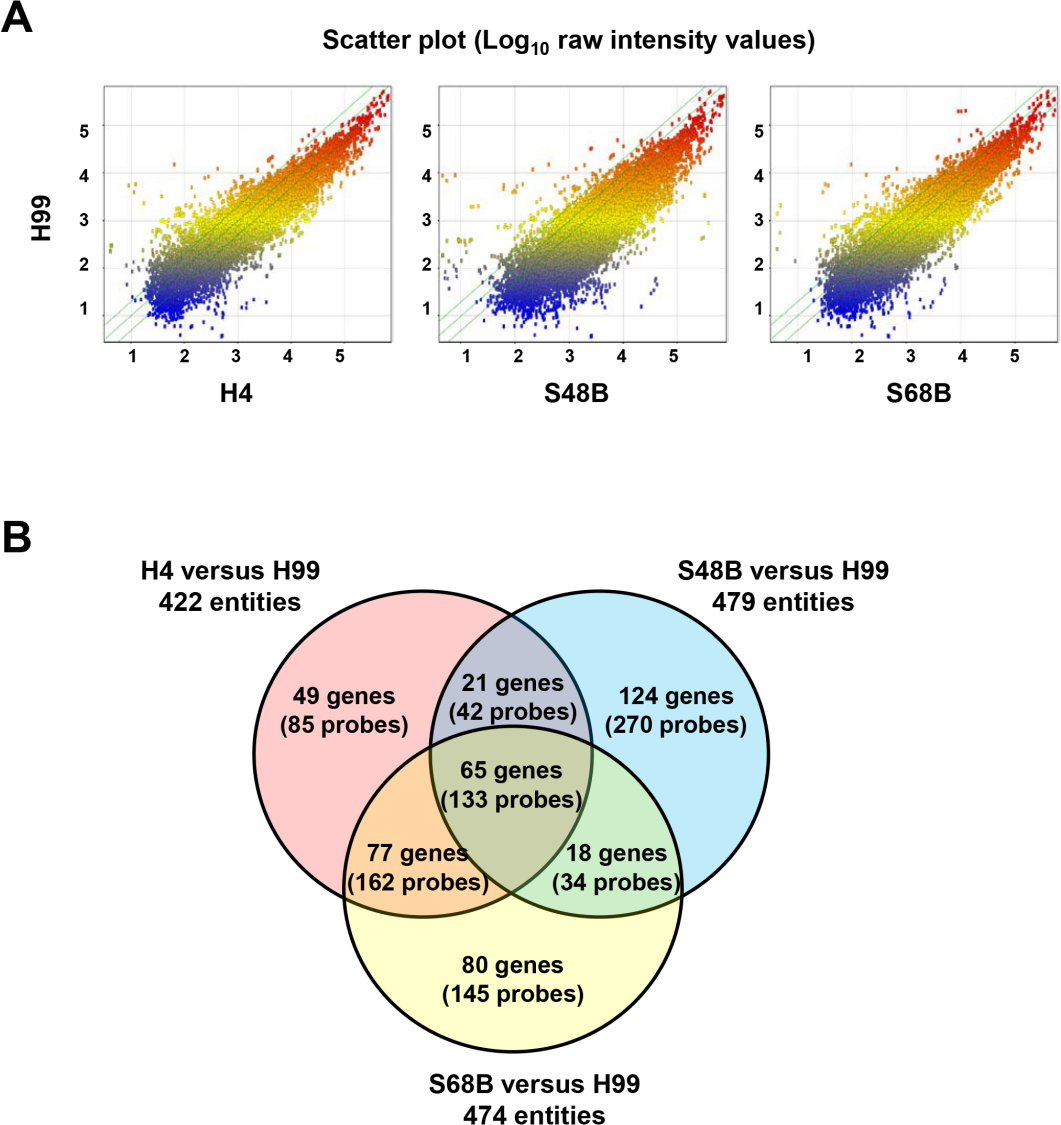
Gene expression of H99 versus environmental strains. (A) Scatter plots show the expressions of total probes in the H99 versus H4, S48B or S68B. X and Y axis show log_10_ raw intensity values. (B) Venn diagram. Significantly regulated probes (FC>2 or FC<-2, P<0.05) were selected from the comparisons of H99 versus each environmental strain. Venn diagram shows the distribution and the number of the genes (probes) in each fraction.

Scatter plots were used to compare the gene expression of H4, S48B and S68B with H99 strain (Fig 4A). Majority of the plots were scattered around the medium line, indicating that no differential expressions between two strains. Those plots exceeding upper and lower border lines were determined as up- or down-regulated probes. A Venn diagram was schemed using significantly expressed genes (FC<-2 or >2, *P*<0.05) to show the distribution of the differentially regulated probes/genes in each group of comparisons (Fig 4B). Some overlapping genes were detected between the strains. Remarkably, 133 probes (65 genes) were overlapped among all groups of comparisons, which suggest that these 65 genes were differentially expressed in the H99 strain relative to all H4, S48B and S68B environmental strains. These genes may have association with the virulence of *Cryptococcus* since H99 strain demonstrated stronger virulence in the intranasal infection of C57BL/6 mice. A list of 65 significant genes was as shown in Table 2.

**Table 2:** 65 significant up- and down-regulated genes in *C*. *neoformans* H99 relative to non-virulent strains. ^#^No hit were obtained using *C*. *neoformans* mRNA sequence in NCBI blast database and a closest hit from *Saccharomyces cerevisiae* was selected. Only known genes or genes which matched blast database are described. vs: versus. The *P* value is determined by an ANOVA analysis, which compares among all sample pairs.

ProbeName	Blast_Hits	Yeast Gene	Yeast Description	H4 vs H99	S48B vs H99	S68B vs H99	*P* value
			Up-regulated genes in H99				
CNAG_03084T0_334	NP_012213^#^	*MMF1*	Protein *MMF1*, mitochondrial	336.6	133.1	453.4	0.0008
CNAG_06956T0_399	NP_192919.1	*MVA1*	Hydroxymethylglutaryl-CoA synthase	318.5	36.1	1151.7	0.0017
CNAG_04901T0_584	NP_013457.1^#^	*BUD8*	Bud-site-selection protein 8	40.5	37.5	62.5	0.0267
CNAG_03433T0_1016				15.3	15.4	10.7	0.0116
CNAG_03772T0_1438	NP_010087	*SNF3*	High-affinity glucose transporter *SNF3*	15.2	8.0	5.5	0.0113
CNAG_03477T0_50	NP_010667.1	*RGA2*	Rho GTPase-activating protein 2	13.6	7.2	11.9	0.0150
CNAG_05426T0_545	NP_013064.1^#^	*PRP19*	E3 ubiquitin-protein ligase PRP19	13.1	5.2	10.2	0.0072
CNAG_01995T0_522	NP_013452.1^#^	*DIC1*	Mitochondrial dicarboxylate transporter	11.6	12.1	13.4	0.0004
CNAG_00826T0_1147	NP_013641	*DAK1*	Dihydroxyacetone kinase 1	11.5	2.6	10.7	0.0006
CNAG_06337T0_1243	NP_010872.1^#^	*GDA1*	**Guanosine-diphosphatase**	7.6	17.5	6.3	0.0020
CNAG_07387T0_1849	NP_011816	*ARN2*	Siderophore iron transporter ARN2	6.7	10.2	11.4	0.0109
CNAG_04507T0_826				6.4	4.1	6.6	0.0018
CNAG_01494T1_640				6.0	5.3	5.2	0.0011
CNAG_03400T0_1015	NP_014490	*GRE2*	NADPH-dependent methylglyoxal reductase GRE2	5.9	3.7	7.9	0.0009
CNAG_01498T0_1754	NP_013090.1^#^	*SOF1*	Protein SOF1http://www.ncbi.nlm.nih.gov/blast/Blast.cgi-alnHdr_6323018	5.7	7.7	8.1	0.0053
CNAG_02011T0_1027	NP_010883.3^#^	*SPF1*	Ion-transporting P-type ATPase SPF1	5.4	2.6	5.0	0.0019
CNAG_07428T0_625	NP_015525.1^#^	*ARR1*	**Arsenicals resistance**	4.8	6.1	5.9	0.0165
CNAG_02083T0_1719	NP_011823	*ARN1*	Siderophore iron transporter ARN1	4.7	8.1	5.5	0.0020
CNAG_00838T0_310	NP_009353.1^#^	*AIM1*	**Altered inheritance of mitochondria** protein 1	4.5	3.1	4.7	0.0141
CNAG_04967T0_1021	NP_011608.1^#^	*VAS1*	Valine-tRNA ligase	4.5	4.2	3.7	0.0156
CNAG_06653T1_22	NP_014127.1^#^	*SEC2*	**Rab guanine nucleotide exchange factor**	4.0	3.3	6.6	0.0046
CNAG_04541T0_1287	NP_011471	*RNA15*	mRNA 3'-end-processing protein RNA15	3.9	3.6	4.0	0.0016
CNAG_06411T0_1614	NP_116685.1^#^	*PTR3*	SPS-sensor componenthttp://www.ncbi.nlm.nih.gov/blast/Blast.cgi-alnHdr_14318552	3.8	4.9	4.9	0.0036
CNAG_04618T1_2110	NP_010111	*UGA3*	Transcriptional activator protein UGA3	3.8	3.0	3.3	0.0226
CNAG_05644T0_925	NP_012683	*YJR149W*	Putative 2-nitropropane dioxygenase	3.1	2.7	3.9	0.0001
CNAG_03393T0_741	NP_012553	*TES1*	Peroxisomal acyl-coenzyme A thioester hydrolase 1	3.0	4.6	5.3	0.0031
CNAG_02439T0_493	NP_009974.1^#^	*CSG2*	Mannosylinositol phosphorylceramide synthase regulatory subunit	3.0	3.2	2.7	0.0001
CNAG_02439T1_409	NP_009974.1^#^	*Rrt12p*	Subtilase type proteinase	3.0	3.2	2.6	0.0002
CNAG_00484T0_1473				2.9	3.0	2.4	0.0186
CNAG_04346T0_968				2.9	3.4	2.9	0.0076
CNAG_06540T1_1679	NP_012550	*ILV3*	Dihydroxy-acid dehydratase, mitochondrial	2.8	2.0	3.9	0.0038
CNAG_04122T0_1086	NP_013755	*ARA2*	D-arabinose 1-dehydrogenase	2.7	2.7	2.4	0.0100
CNAG_02336T0_1615	NP_013031	*VBA5*	Vacuolar basic amino acid transporter 5	2.6	2.6	4.7	0.0096
CNAG_07980T0_45	NP_010066.1^#^	*GDH2*	Glutamate dehydrogenase (NAD(+))	2.5	2.4	3.2	0.0034
CNAG_04034T0_839	NP_013086.1	*BPT1*	ATP-binding cassette bilirubin transporter BPT1	2.4	2.2	3.1	0.0123
CNAG_03819T0_544	NP_013706	*ERG6*	Sterol 24-C-methyltransferase	2.4	2.7	3.2	0.0187
CNAG_04363T0_2449	NP_011853.1^#^	*ETP1*	Ring finger proteinhttp://www.ncbi.nlm.nih.gov/blast/Blast.cgi-alnHdr_6321777	2.4	2.5	3.0	0.0199
CNAG_06239T0_1499	NP_013580.1	*ERG13*	Hydroxymethylglutaryl-CoA synthase	2.2	2.7	2.2	0.0151
CNAG_06806T0_862	NP_015329	*AIM45*	Probable electron transfer flavoprotein subunit alpha, mitochondrial	2.1	2.7	2.3	0.0078
CNAG_05784T1_1057	NP_012234	*SSM4*	E3 ubiquitin-protein ligase Doa10	2.1	2.0	2.2	0.0008
			**Down-regulated genes in H99**				
CNAG_05332T0_622	NP_012694	*MPH2*	Alpha-glucosides permease MPH2/3	57.4	189.8	69.0	0.0001
CNAG_05333T0_1673	NP_012910	*PUT3*	Proline utilization trans-activator	51.3	69.7	32.0	0.0179
CNAG_03087T0_1523	NP_009857	*MAL31*	Maltose permease MAL31; Maltose permease MAL61	44.8	13.6	23.3	0.0064
CNAG_01858T0_1311	NP_010021	*HMRA1*	Mating-type protein A1	21.3	17.6	12.1	0.0086
CNAG_07313T0_320				16.2	151.2	16.3	0.0003
CNAG_04030T0_242	NP_014029.1	*ADE4*	amidophosphoribosyltransferase	12.6	5.8	5.0	0.0117
CNAG_06868T0_730	NP_012044	*ENO2*	Enolase 2	11.2	9.4	15.3	0.0063
CNAG_07707T0_1685	NP_015447.1^#^	*AXL1*	Putative protease	7.2	4.9	9.4	0.0068
CNAG_05973T0_1159	NP_013769.1^#^	*STB2*	Sin3 complex subunit	6.2	15.8	8.2	0.0109
CNAG_05872T0_1058	NP_015171	*PEP4*	Saccharopepsin	6.1	2.5	4.6	0.0014
CNAG_04185T0_667	NP_015414.2^#^	*YPR089W*	hypothetical protein YPR089W	5.4	3.5	4.8	0.0158
CNAG_06016T0_1557	NP_012789.2^#^	*YKL133C*	hypothetical protein YKL133C	5.2	5.9	3.8	0.0044
CNAG_02992T0_2056	NP_010414	*SAC6*	Fimbrin	5.0	5.0	4.8	0.0303
CNAG_05786T1_1058	NP_015338	*YPR013C*	Zinc finger protein YPR013C	4.6	2.4	3.6	0.0037
CNAG_02351T0_1732				3.8	3.9	4.4	0.0145
CNAG_04978T0_794	NP_011884.1^#^	*YHR020W*	proline—tRNA ligase	3.7	4.2	3.5	0.0233
CNAG_06334T0_1554	NP_009405.1^#^	*SEN34*	tRNA-splicing endonuclease subunit	3.6	2.6	3.2	0.0037
CNAG_01533T0_2123	NP_015210	*BEM3*	GTPase-activating protein BEM3	3.4	5.2	3.4	0.0004
CNAG_04737T0_2461	NP_013895.1^#^	*HOT1*	Highosmolarity-induced transcription protein 1	3.2	5.3	5.5	0.0046
CNAG_06359T0_714	NP_013372	*DCS1*	Scavenger mRNA-decapping enzyme	3.1	3.0	2.3	0.0130
CNAG_03136T0_1325				2.8	2.3	2.8	0.0166
CNAG_01082T0_3293	NP_010550	*AKR1*	Palmitoyltransferase AKR1	2.6	2.4	2.9	0.0240
CNAG_06223T0_1780	NP_014799	*NFI1*	E3 SUMO-protein ligase	2.5	2.1	2.2	0.0013
CNAG_03689T0_1787	NP_013860	*SIP5*	Protein SIP5	2.5	2.4	2.4	0.0268
CNAG_07939T0_65				2.1	2.6	3.1	0.0084

### 3.4. Significant up-regulated genes in *C*. *neoformans* H99 compared to non-virulent strains

**Fig 5:**
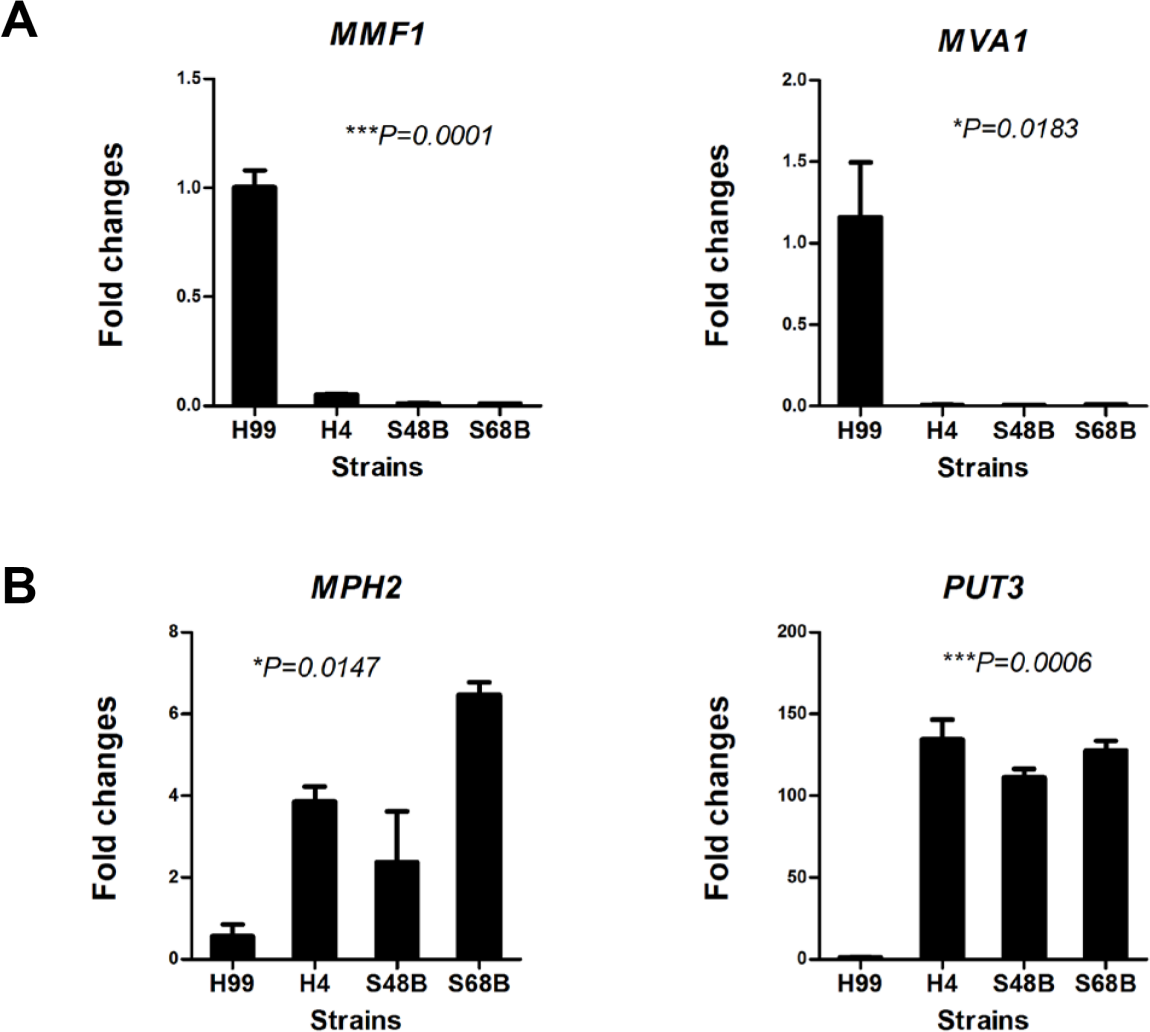
qRT-PCR verification. RNAs were extracted from H99, H4, S48B and S68B for qRT-PCR analysis. Two independent samples were prepared for each fungal strain. All samples were run in triplicates and data were shown as mean ± SD. Group statistical significance measured was by one-way ANOVA analysis (*P<0.05, **P<0.01, ***P<0.001).

Among top of the genes that were expressed at an intensely high level in *C*. *neoformans* H99 relative to environmental strains included *MVA1*, *MMF1*, *BUD8*, *SNF3* and *RGA2*. In contrast, genes that showed significant lower expression in H99 strain included *MPH2*, *PUT3*, *MAL31*, *HMRAI* and *ENO2*. The potential functions for significantly up-regulated genes in causing fungal virulence are discussed in the Discussion. To validate the microarray data, expression of selected top differentially regulated genes including *MVA1*, *MMF1*, *MPH2* and *PUT3* were verified using qRT-PCR (Fig 5). Consistent with the microarray data, qRT-PCR showed that expression levels of *MVA1* and *MMF1* were significantly increased in H99 relative to other environmental strains at >142 and >19 folds, respectively. On the other hand, the *MPH2* and *PUT3* were reduced in H99 at >4.2 and >25 folds, respectively.

### 3.5. Pathway analysis

**Fig 6:**
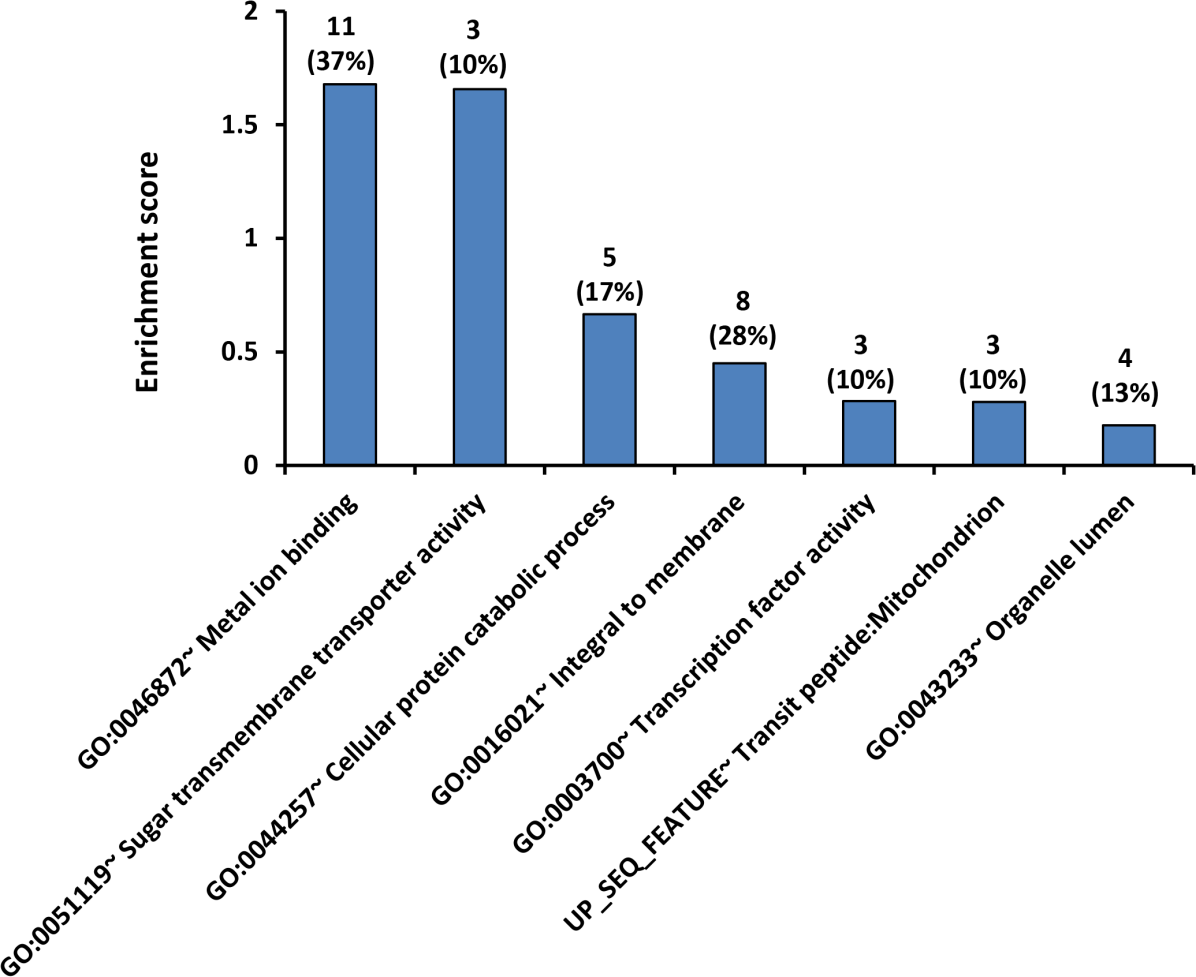
Pathway analysis. Histogram shows a summary of DAVID pathway annotation analysis. The 65 genes which were commonly regulated in H99 versus three environmental strains were selected and analyzed. The number of significant genes and their percentages of genes in the database are as indicated.

The over-represented pathway categories of the 65 significant genes in H99 strain were analyzed using DAVID bioinformatics resource (Fig 6). Representative annotation of each cluster and gene details were as listed in Table 3. The top in the list of the over-represented pathways was metal ion binding (GO:0045872) with enrichment score (ES) at 1.68 (*P* = 0.015). A total of eleven genes (out of the 65 significant genes) were found to be listed in the metal ion binding pathway database, suggesting that considerable numbers of the H99-specific genes were associated with metal ion binding process (Table 3). In addition, three genes were found to be associated with sugar transmembrane transporter activity in fungal cells (GO:0051119) (ES = 1.65, *P* = 0.010). Other relevant pathways included cellular catabolic process (GO:0044257), integral to membrane (GO:0016021), transcription factor activity (GO:0003700), transit peptide:mitochondrion (Up_Seq) and organelle lumen (GO:0043233), however these five pathways were not statistically significant.

**Table 3:** Pathway annotation by DAVID bioinformatics resource. The 65 significant genes (133 overlapped probes) which were differentially regulated in H99 compared to environmental strains were analyzed. Shown were representative of each annotation cluster detected. Count represents number of genes which match the pathway database, and % represents the percentage of gene hits among the total genes in the pathway database. Enrichment score (ES) of each group was measured by the geometric mean of the EASE Scores (modified Fisher Exact) associated with the enriched annotation terms that belong to this gene group. Population hit (Pop Hits) represents how many have the function name in your gene list of interest, and population total (Pop Total) represents how many genes in overall population has that function name in the background genome (all genes in the species of interest in DAVID database). False discovery rate (FDR) represents the percentages of test which might be false positive. *P* values were analyzed using Fisher exact score to identify which sub-populations are over- or under-represented in a sample. Data were considered significant if *P<0.05.

Category	Term	Genes	ES	Count	%	Pop Hits	Pop Total	FDR	*P*
GOTERM_MF_FAT	GO:0046872~ Metal ion binding	*UGA3*, *SSM4*, *PUT3*, *YPR013C*, *SAC6*, *ENO2*, *AKR1*, *ARN1*, *ILV3*, *ARN2*, *NFI1*	1.67	11	37.9	830	4190	16.7	0.0159*
GOTERM_MF_FAT	GO:0051119~ Sugar transmembrane transporter activity	*MAL31*, *SNF3*, *MPH2*	1.65	3	10.3	26	4190	10.9	0.0101*
GOTERM_BP_FAT	GO:0044257~ Cellular protein catabolic process	*SIP5*, *PEP4*, *SSM4*, *DCS1*, *NFI1*	0.66	5	17.2	351	4870	82.9	0.1255
GOTERM_CC_FAT	GO:0016021~ Integral to membrane	*SSM4*, *MAL31*, *SNF3*, *MPH2*, *AKR1*, *VBA5*, *ARN1*, *ARN2*	0.44	8	27.5	1488	4595	99.7	0.4214
GOTERM_MF_FAT	GO:0003700~ Transcription factor activity	*UGA3*, *PUT3*, *HMRA1*	0.28	3	10.3	135	4190	91.1	0.1917
UP_SEQ_FEATURE	UP_SEQ_FEATURE~ Transit peptide:Mitochondrion	*AIM45*, *MMF1*, *ILV3*	0.27	3	10.3	325	6448	99.7	0.415
GOTERM_CC_FAT	GO:0043233~ Organelle lumen	*PEP4*, *AIM45*, *RNA15*, *MMF1*	0.17	4	13.7	783	4595	99.9	0.6522

## Discussion

The mice virulence study showed that the fungal loads in both lung and brain tissues of H99-infected mice were significantly higher relatively to the mice infected with Malaysian environmental strains (S48B, S68B or H4). The environmental strains of *C*. *neoformans* can be found ubiquitously in the soil, certain trees and bird guano. Most of the people are exposed to *C*. *neoformans* during childhood through inhalation because of the ubiquitous existence of environmental strains [27]. However, a previous study suggests that most of the environmental strains isolated are non-lethal to the mice and only one out of eleven strains tested is virulent [26]. Our study showed that H99 clinical strain was highly virulent in mice, while S48B and S68B strains were less virulent with detectable numbers of CFU in lung and brain homogenates. The H4 strain was considered non-virulent as no CFU could be observed in both tissue homogenates. Because the four strains used in this study were all *C*. *neoformans* serotype A, molecular type VNI, and shared ~93% genetic similarities (Supporting Information S1 Fig) [18], the mechanism underlying their difference degrees of infectivity remains elusive. We began this study by hypothesizing that this difference could plausibly be a resultant of their distinct gene transcriptional programs, which renders invasive or non-invasive characteristics. Microarray data analysis was conducted to substantiate our hypothesis.

Pathway annotation analysis using DAVID bioinformatics resource revealed two significantly pathways i.e. metal ion intake (ES = 1.67, *P* = 0.0159) and sugar transmembrane transporter activity ES = 1.65, *P* = 0.0101). This finding suggests that the ability of metal ion uptake and sugar transmembrane transport might distinct between the virulent strain H99 and less/non-virulent environmental strains. Transition metals such as iron, zinc, copper, and manganese are essential elements for the growth and survival of microorganisms including fungi. These microorganisms sequester the metal ions from the host cells through expressing high affinity metal ion transporters to import the nutrient ions [28]. Evidences suggest that metal ion intake is involved in microbial pathogenesis. Iron uptake can regulate the transcription and capsule formation in *C*. *neoformans* [29] thus contribute to the fungal cell virulence. In fact, pulmonary infection using *C*. *neoformans* mutant strains lacking or defective in copper coordination exhibit reduced pulmonary colonization [30]. It has been reported that copper was required to restore the activity of defective laccase in a *DVPH1* avirulent mutant of *C*. *neoformans* [31]. Hence, low expression of the environmental strains in metal ion uptake may be a cause for the lower laccase activities of our environmental strains compared to the clinical strains, as noted in a previous study [32]. Besides, mutations in *Nickel transporter gene NIC1* and urease proteins attenuate the invasion of *C*. *neoformans* into mice central nervous system [33]. Up-regulation of *Siderophore iron transporter ARN/SIT*-associated genes are discussed below.

Another significant pathway identified was sugar transmembrane transport. *C*. *neoformans* is an encapsulated yeast with sugar-coating, in which the sugar transport regulators are essential for the production of capsular polysaccharide antigens [34]. In fact, top in the list of the up-regulated genes in the H99 comprised *SNF3*, a gene which is linked to the sugar transmembrane transport [35, 36]. Our data showed that *SNF3* was up-regulated at 15.2-, 8.0- and 5.5-fold in H99 versus H4, S48B and S68B strains. Mutations in the yeast *SNF3* gene affect glucose sensing and *SNF3* mutants show defective growth on glucose [37]. In the absence of *SNF3*, yeast cells show reduced lifespan and caloric restriction effectiveness due to impaired mitochondrial activities [38].

Microarray data analysis showed that the expression profile of H99 differs from the rest of the environmental strains. We managed to identify a list of 65 genes that were commonly up- or down-regulated in H99 compared to H4, S48B or S68B strains. Referring to the variances of the degree of virulence among H99 and environmental strains H4, S48B and S68B, we hypothesized that the differentially regulated genes in H99 likely play roles in determining the fungal virulence. The main problem encountered in the data analysis was that not all *C*. *neoformans* genes have been fully characterized and reported. For certain genes, BLAST was carried out to find the closest matched genes from widely studied species i.e. *Saccharomyces cerevisiae*. Functional prediction for many genes was also reviewed based on the previous studies carried out using *S*. *cerevisiae* or *Candida albicans*. No suitable matches were found for some of the genes reported in Table 2.

The highest up-regulated gene in the H99 strain was *Mitrochondrial matrix factor* (*MMF1*), which was up-regulated at 336.6-, 133.1- and 453.4-fold in the *C*. *neoformans* H99 strain versus other environmental strains. *MMF1* is responsible in the maintenance of intact mitochondria [39, 40]. Mitochondrial function is essential for metabolic pathways such as the glyoxylate cycle and gluconeogenesis as well as in sustaining cell survival throughout oxidative stress [41, 42]. In addition, the mitochondrial function in membrane lipid homeostasis confers drug tolerance ability in fungus [43]. *C*. *albicans* mutants which lack *GOA1*, a mitochondrial protein or subunits of the mitochondrial pyruvate dehydrogenase complex, show a defect in yeast-to-filamentous switching and impairment in systemic invasion [44, 45]. Besides, the hyper virulence *C*. *gattii* isolated during the outbreaks in Vancouver Island and North America [16] has been associated with more efficient mitochondrial function [46]. In addition to *MMF1*, four other mitochondrial associated genes were found among the 65 significant genes in H99. These genes were *Mitochondrial dicarboxylate transporter DIC1*, *Altered inheritance of mitochondria protein 1 AIM1*, *Mitochondrial Dihydroxy-acid dehydratase ILV3* and *Mitochondrial probable electron transfer flavoprotein subunit alpha AIM45*, which were induced at >2-fold (*P*<0.05) in the H99 compared to environmental strain, suggesting the active involvement of mitochondrial activity in driving fungal virulence.

Another highly up-regulated gene was a novel gene with unpredicted function, which matches to *S*. *cerevisiae MVA1* at 53% gene similarity in the NCBI database. *MVA1* was upregulated at 318.5-, 26.1-, and 1151.7-fold in the H99 stain versus H4, S48B and S68B environmental strains. *MVA1* is a acetyl-CoA C-acetyltransferase (also named as hydroxymethylglutaryl-CoA synthases) which changes the acetyl-CoA into hydroxymethylglutaryl-CoA in the cholesterol synthesis pathway. In fungi, acetyl-CoA is important for the synthesis of cell wall chitin and O-acetylation of the capsule [47]. Acetylation of capsule polysaccharide enables inhibition of neutrophil migration and suppression of host immune response [48]. Importantly, elevated expression of acetyl-CoA production and utilization-associated genes has been detected in *C*. *neoformans* recovered from the lung of infected mice during pulmonary infection [47].

Another significant up-regulated gene identified in this study, *Bud-site-selection protein 8* (*BUD8*), was induced at 40.5-, 37.5- and 62.5-fold. *BUD8* is a transmembrane glycoprotein which participates in the proteins complex to organize and polymerize actin and actin-associated proteins [49, 50]. The function of *BUD8* is to ensure bipolar budding during cell division and polarized growth [51], important for bud initiation during invasion process [52]. Besides, another gene in the top list, *RGA2*, induced at 13.6-, 7.2- and 11.9-fold in H99 compared to environmental strains, was also associated with yeast budding process. *RGA2* is a Rho GTPase-activating protein (GAP) of the central polarity regulator CDC42 that functions to regulate cell morphogenesis and integrity [53]. In *C*. *albicans*, hyperphosphorylation of *RGA2* occurs at the bud emergence and governs different forms of polarized morphogenesis [54]. The functions of *BUD8* and *RGA2* in *C*. *neoformans* and their roles in fungal virulence require further investigations.

Other selected genes that were considerably up-regulated and have a reported role in virulence were as discussed below. For instance, two genes in Siderophore iron transporters family, *ARN2* and *ARN1* were up-regulated at 6.7-, 10.2-, and 11.4-fold, and 4.7-, 8.1-, and 5.5-fold, respectively, in H99 when compared to H4, S48B and S68B strains. Non-reductive uptake of ferric–siderophore complexes is dependent on specific siderophore transporters *ARN1*, *ARN2*, *ARN3* and *ARN4* [55, 56]. Some pathogenic fungi, such as *S*. *cerevisiae*, have homologues for siderophore transporters that help these fungi to utilize siderophores from competitors [55]. *C*. *albicans ARN1/SIT1* is essential for the fungal epithelial cell invasion [57–59]. Whereas in *Aspergillus fumigatus*, loss of ability to synthesize siderophores appears to compromise its virulence [29, 60–62]. Recently, the transcript for a putative siderophore transporter *ARN1/SIT1* which was increased in *C*. *neoformans* cells grown in low-iron medium, has been characterized [63]. Therefore, *ARN1* and *ARN2* may contribute to the fungal cell virulence by controlling the ability of *C*. *neoformans* to acquire siderophores.

*Guanosine-diphosphatase* (*GDA1*), a GDPase which functions to hydrolyze GDP to GMP, was induced at 7.6-, 17.5- and 6.3-fold in H99 relative to H4, S48B and S68B. The adhesive and immunomodulation properties of some fungal pathogens depend on cell wall mannoproteins [64]. *GDA1* hydrolyzes the GDP-mannose complexes thus releases mannans to the cell wall. In *GDA1*-mutated *C*. *albicans*, defect in GDP hydrolysis, O-mannosylation can also result in impaired yeast-hypha transition [65]. Another up-regulated gene, *Ino-transporting P-type ATPase* (*SPF1*) was increased at 5.4-, 2.6- and 5.0-fold in H99 versus environmental strains. *SPF1* plays essential role in calcium homeostasis, and its deletion results in calcium influx and increased cellular calcium contents, leading to expression of the calcium-dependent response elements gene *CCH1* for the cell survival [66]. Besides, *SPF1* null mutant shows defects in hyphal growth rate and biofilm formation, resulting in severely attenuated virulence in *C*. *albicans* [67].

*NADPH-dependent methylglyoxal reductase* (*GRE2*) was increased 5.9-, 3.7- and 7.9-fold in H99 versus H4, S48B and S68B strains. The cAMP-dependent genes *GRE2* in *C*. *neoformans* is known to be induced by a variety of environmental stresses, including osmotic and oxidative stresses [68]. In addition, *GRE2* involves in metabolism of ergosterol [69], a V-ATPase which functions to repress growth and attenuate fungal virulence in both *S*. *cerevisiae* and *C*. *albicans* [70]. On the other hand, *NAD(+)-dependent Glutamate dehydrogenase* (*GDH2*) was induced at 2.5-, 2.4- and 3.2-fold. GDH2 degrades the reversible oxidative deamination of glutamate to α-ketoglutarate and ammonia using NAD(H) and NADP(H) as cofactors. It also interacts with *GDH3* and suppresses stress-induced apoptosis in the cells [71]. Therefore, high expression of *GRE2* and *GDH2* in H99 may influence cell virulence by regulating biochemistry pathways in *C*. *neoformans*.

*Rab guanine nucleotide exchange factor* (*SEC2*) was induced at 4.0-, 3.3-, 6.6-fold in H99 compared to H4, S48B and S68B. *SEC2* functions in polarized directional delivery of post golgi vesicle for exocytosis activity [72, 73]. Phosphorylated form of *SEC2* binds preferentially to *SEC15* and a component of exocyst tethering complex which enables fusion of secretory vesicle with plasma membrane [74]. The phosphorylation of *SEC2* is necessary to support hyphal in *C*. *albicans* because during hyphal extension, the distance of hyphal tip and nucleus is far, thus an effective transport machinery through *SEC2* is required [75].

*Dihydroxyacetone kinase 1* (*DAK1*) was found to be up-regulated in H99 for 11.5-, 2.6- and 10.7-fold. *DAK1* functions to catalyze both phosphorylation of dihydroxyacetone (DHA) of glyceraldehyde. In *S*. *cerevisiae*, detoxification of dihydroxyacetone by *DAK1* is suggested to be a vital part of the physiological response during diverse stress conditions [76]. Interestingly, *DAK* has been found to be an interacting protein for *MDA-5*, a pattern recognition receptor on host immune cells. *DAK* interacts with *MDA-5* and blocks its antiviral signaling [77]. The overexpression of *DAK* inhibits *MDA5*-mediated IFN-β induction and cytoplasmic dsRNA-/virus-induced activation of the IFN-β promoter, whereas these processes can be increased by RNAi knockdown of endogenous *DAK*. It remains to be investigated if the *DAK1* expressed in *C*. *neoformans* H99 can inhibit *MDA5*-mediated signaling to suppress host immune response.

Although comparative gene transcriptome profile can provide us valuable information and prediction of potential virulence genes, further investigations on *C*. *neoformans* have shown that gene regulation does not always correlate with their functional activities. For example, *C*. *neoformans Isocitrate lyase* (*ICll*) gene is highly upregulated during central nervous system infection nonetheless the null mutant for *ICll* does not exhibit an attenuated phenotype *in vivo* [7]. Therefore, mutagenesis studies of the genes discussed are needed in future study to confirm their functions and roles in fungal pathogenesis.

## Supporting Information

S1 Fig UPGMA algorithm based-dendrogram shows the cluster analysis of four *C*. *neoformans* strains. Genomic DNA was isolated using MasterPure Yeast DNA Purification Kit (Epicentre, USA) and checked using a nanophotometer (Implen, Germany). PCR fingerprinting was then performed as previously described [18]. Briefly, genomic DNA was amplified using primer (GTG)_5_: 5’-GTGGTGGTGGTGGTG-3’ primers. PCR products were separated on 2.0% agarose gels electrophoresis and visualized under UV light. Digital images were further analyzed by the PyElph software. Cluster analysis of the cryptococcal isolates was performed with the unweighted-pair-group method using arithmetic averages (UPGMA).
(TIF)
