# Supplementary figures and images for: Genome-Wide Transcription Study of Cryptococcus neoformans H99 Clinical Strain versus Environmental Strains
Source: PLoS One. 2015 Sep 11;10(9):e0137457. doi: 10.1371/journal.pone.0137457 (PMC4567374; doi:10.1371/journal.pone.0137457)

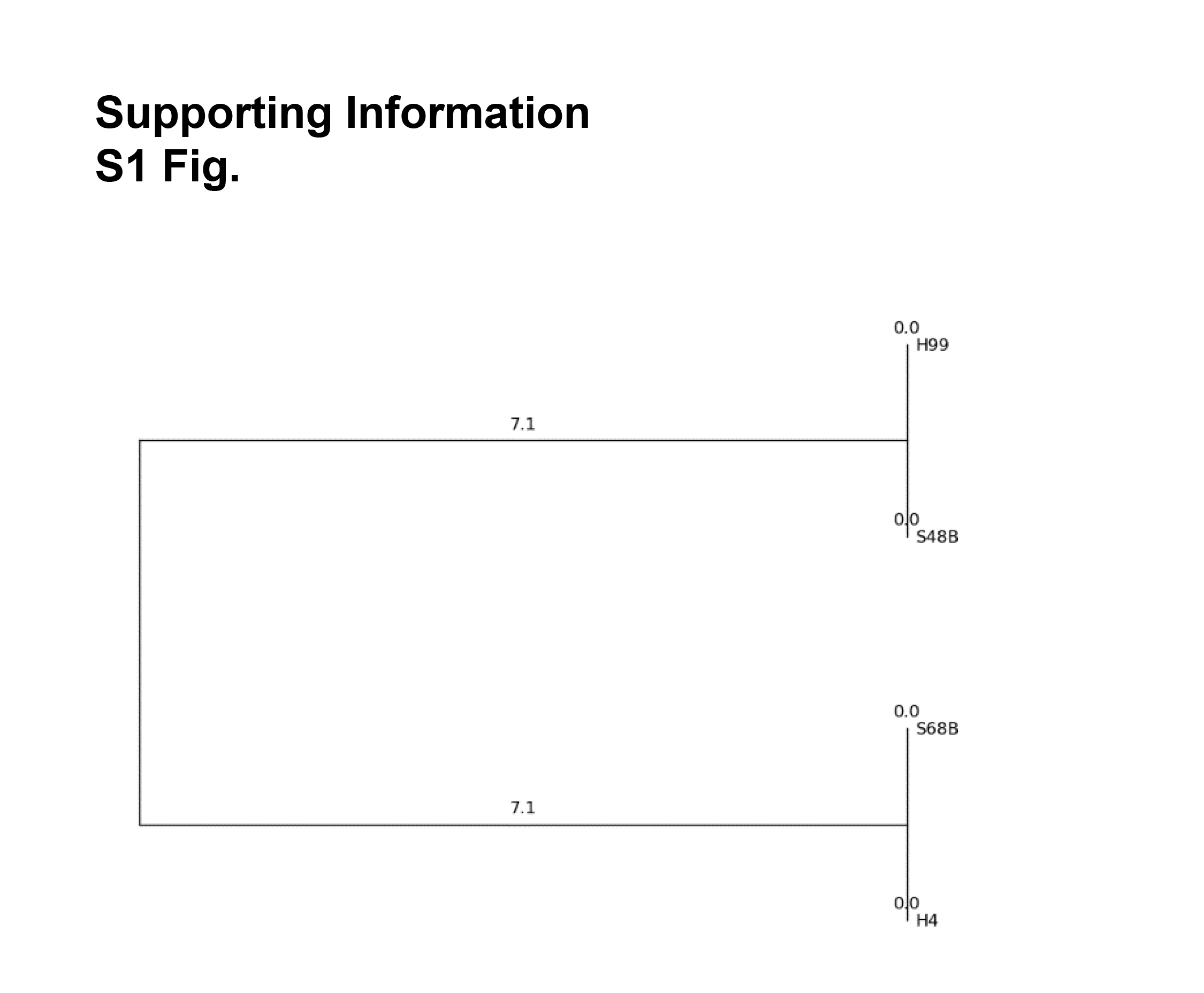

Supplement: S1 Fig — Genomic DNA was isolated using MasterPure Yeast DNA Purification Kit (Epicentre, USA) and checked using a nanophotometer (Implen, Germany). PCR fingerprinting was then performed as previously described [18]. Briefly, genomic DNA was amplified using primer (GTG)5: 5’-GTGGTGGTGGTGGTG-3’ primers. PCR products were separated on 2.0% agarose gels electrophoresis and visualized under UV light. Digital images were further analyzed by the PyElph software. Cluster analysis of the cryptococcal isolates was performed with the unweighted-pair-group method using arithmetic averages (UPGMA). (TIF) [file pone.0137457.s001.tif]
